# Exploratory Evaluation of Cranial Suture Lengths As Supplementary Indicators of Stature: An Autopsy-Based Morphometric Study in a North Indian Population

**DOI:** 10.7759/cureus.109755

**Published:** 2026-05-27

**Authors:** Mustafa Khan, Hitesh Chawla, Kapil Yadav, Aravindan U., Nayan Gautam, Nasir Khan

**Affiliations:** 1 Forensic Medicine & Toxicology, Shaheed Hasan Khan Mewati Government Medical College, Nalhar, IND; 2 Forensic Medicine &amp; Toxicology, Maharishi Chyawan Medical College, Koriawas, IND; 3 Forensic Medicine &amp; Toxicology, All India Institute of Medical Sciences, Madurai, IND

**Keywords:** anthropometry, autopsy, cranial sutures, identification, stature

## Abstract

Background: Stature estimation is an important component of forensic identification, particularly when incomplete skeletal remains are encountered. Although long bones are the preferred skeletal elements for stature estimation, cranial parameters may provide supplementary information when conventional skeletal remains are unavailable. The potential relationship between cranial suture length and stature remains insufficiently explored. This exploratory study aimed to evaluate possible associations between cranial suture lengths and stature and to assess their potential supplementary role in stature estimation in a North Indian population.

Materials and methods: This prospective cross-sectional autopsy-based study included 80 medico-legal autopsy cases aged ≥21 years. Coronal, sagittal, and lambdoid suture lengths (CSL, SSL, and LSL) were measured ectocranially using a thread-and-caliper method. Supine body length was recorded as stature. Pearson correlation and linear regression analyses were performed to evaluate associations between suture lengths and stature. Regression assumptions were assessed prior to model interpretation.

Results: The study included 80 individuals (54 men and 26 women) aged 21-80 years. Mean stature in male subjects was 1698.7 ± 80.9 mm, whereas in female subjects, it was 1585.4 ± 92.9 mm, with a statistically significant difference between sexes (p = 0.0005). SSL and LSL demonstrated statistically significant but weak positive correlations with stature in the combined sample (r = 0.302 and 0.297, respectively). CSL showed no statistically significant association. Sex-specific analyses did not demonstrate significant correlations. Linear regression analysis produced the following equations for stature estimation:

Stature (mm) = 1356.151 + 2.219 × SSL (mm)

Stature (mm) = 1401.116 + 1.506 × LSL (mm)

Regression models demonstrated low explanatory power (R² < 0.10), indicating limited predictive capability.

Conclusion: Cranial suture lengths demonstrated statistically significant but weak associations with stature in the combined study population. However, the low predictive accuracy and limited explanatory power indicate that these measurements possess restricted forensic utility when used independently. Cranial suture measurements should therefore be considered supplementary rather than primary indicators for stature estimation. Further validation studies with larger populations are required.

## Introduction

Human identification is a central objective of forensic investigation and plays a crucial role in medico-legal practice. Identification refers to the determination of the individuality of a person and may be either complete or partial. Complete identification establishes the definite identity of an individual, whereas partial identification involves determining specific biological characteristics such as age, sex, stature, or ancestry. Forensic pathologists frequently encounter mutilated, decomposed, or skeletonized remains in routine medico-legal practice. Such cases may arise from natural disasters, mass casualties, criminal dismemberment, or advanced decomposition. Under these circumstances, establishing identity becomes a challenging task. In many instances, forensic investigators rely on partial identification parameters, including age, sex, and stature, to narrow the pool of potential identities before confirmatory methods such as DNA profiling are applied [1].

Stature estimation constitutes a key component of the biological profile in forensic anthropology [2]. It represents an important biological characteristic that contributes significantly to personal identification [3]. Stature is one of the biological identities that can be estimated from the skeleton even long after the death of an individual [4]. The estimate of stature is not a concern when intact bodies are being examined. However, when dismembered human body parts are the resources to work with, forensic pathologists have an even harder task [3].

An undeniable biological relationship exists between stature and bodily components such as the extremities, head, trunk, and spinal column [5]. Reliable estimation of stature from skeletal elements is generally performed after skeletal maturity has been attained, which usually occurs during the late teenage years or early twenties [6]. In cases involving skeletal remains, forensic anthropology provides important tools for estimating stature from fragmentary skeletal elements. Numerous studies have demonstrated that regression equations derived from intact long bones of the upper and lower extremities provide relatively accurate estimates of stature and are typically population- and sex-specific. However, in situations where only partial skeletal remains are available, particularly cranial remains, the application of traditional long-bone-based regression models becomes impossible. When a skull of unknown origin is found and no other means of identification is possible due to decomposition, then the ability to determine sex, age, race, and stature from the skull is of great value [7].

The human skull contains multiple anatomical landmarks that have been explored for stature estimation, including cranial dimensions, facial measurements, and cephalometric parameters [8,9]. Despite these investigations, the potential role of cranial sutures in stature estimation has received relatively little attention in forensic research. Cranial sutures represent fibrous joints between skull bones that accommodate cranial vault expansion during growth and development. Measurement of cranial sutures is relatively simple and can be performed even when only the skull vault is preserved. Therefore, investigating the relationship between cranial suture length and stature may provide an additional tool for forensic identification in cases involving fragmentary cranial remains.

Although cranial sutures are not directly involved in longitudinal skeletal growth, cranial vault morphology may exhibit indirect associations with overall body size through shared developmental, genetic, and environmental influences. However, the biological relationship between cranial suture length and stature remains poorly understood and insufficiently investigated in forensic anthropology. The present exploratory study aimed to evaluate possible associations between the lengths of coronal, sagittal, and lambdoid cranial sutures and stature in a North Indian population and to assess their potential supplementary role in stature estimation when conventional skeletal parameters are unavailable.

## Materials and methods

This prospective cross-sectional autopsy-based study was conducted in the Department of Forensic Medicine at a tertiary care teaching hospital in southern Haryana, India. The study period extended from April 2023 to April 2024. Ethical approval was obtained from the Institutional Ethics Committee prior to commencement of the study. A total of 80 medico-legal autopsy cases were included consecutively during the study period based on eligibility criteria to minimize selection bias. The study population primarily represented individuals from North Indian regional ancestry as documented in hospital and medico-legal records. Individuals aged 21 years and above with known and verified age were considered eligible. Only cases with intact cranial vaults suitable for examination of cranial sutures were included. Cases involving fractured or deformed skulls, advanced decomposition, charring, or mutilation that could interfere with accurate measurement were excluded. Crania with wormian (sutural) bones were also excluded to avoid potential alteration of suture morphology.

Stature was measured with the body placed in a supine position on the autopsy table with all the joints fully extended, and the head and feet were maintained in a neutral anatomical position. The measurement was taken from the vertex of the head to the base of the heel along the mid-sagittal plane using a measuring tape. Supine body length was recorded in centimeters to the nearest 0.1 cm and subsequently converted to millimeters for statistical analysis.

**Figure 1:**
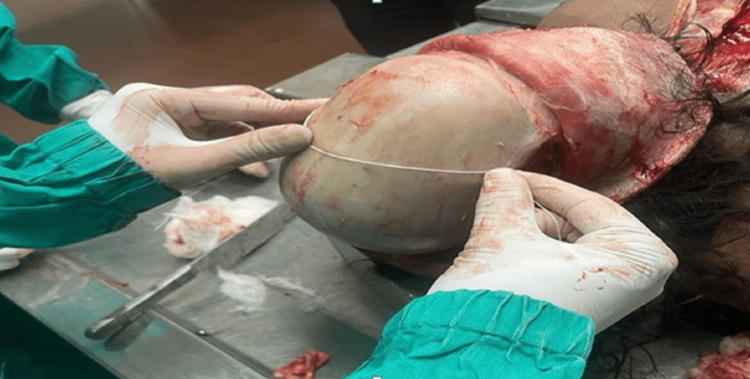
Measurement of CSL CSL: Coronal suture length

Following routine autopsy procedures, the scalp was reflected to expose the cranial vault. The temporal muscles were dissected and the periosteum carefully removed to expose the coronal, sagittal, and lambdoid sutures ectocranially. Suture lengths were measured using a non-extensible thread carefully placed along the entire course of the suture to accommodate its curvature (Figure 1).

**Figure 2:**
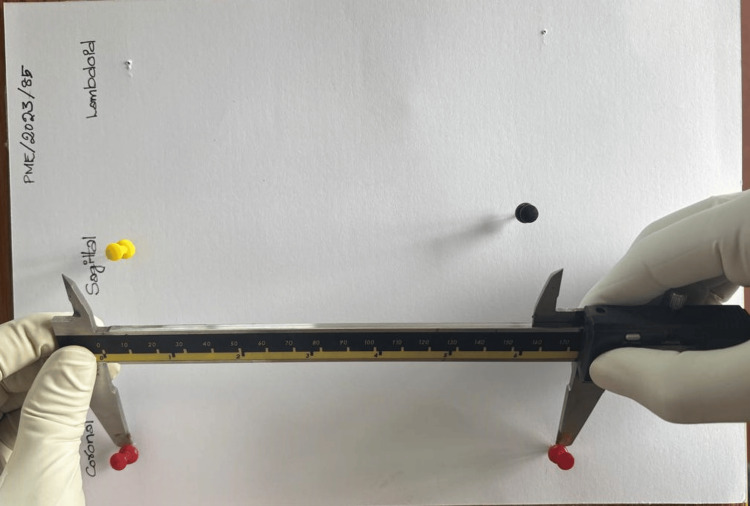
Measurement of suture thread using a Vernier caliper

The thread was subsequently measured using Vernier calipers calibrated in millimeters. The Vernier calipers were calibrated prior to data collection according to manufacturer specifications to ensure measurement accuracy. To ensure measurement accuracy, the thread was secured on a cardboard surface using pins, and the distance between the pins was measured using Vernier calipers (Figure 2).

Each measurement was performed carefully by the same observer to minimize observer error. The measurements were taken thrice and average was considered.The coronal suture length (CSL) was measured from the right pterion to the left pterion. The sagittal suture length (SSL) was measured from the bregma to the lambda. The lambdoid suture length (LSL) was measured from one asterion to the opposite asterion [10]. All anatomical landmarks were identified according to standard osteological definitions to maintain consistency in measurement procedures.

Because the study was conducted on medico-legal autopsy cases, repeat measurement of the cranial sutures on the original specimens after a prolonged interval was not feasible. However, to assess measurement consistency, the thread lengths obtained from selected specimens were re-measured using calibrated Vernier calipers after a two-week interval under identical conditions. The repeated measurements demonstrated acceptable reproducibility.

Data were entered into Microsoft Excel and analyzed using IBM SPSS Statistics Version 29 (IBM Corp., USA). Descriptive statistics were expressed as mean and standard deviation for continuous variables. Independent sample t-tests were used to compare measurements between sexes. Pearson correlation coefficients were calculated to assess the relationship between stature and suture lengths. Linear regression analysis was performed to derive equations for predicting stature. Residual normality was evaluated using Q-Q plots. External or internal validation procedures were not performed because of the limited sample size. A p-value less than 0.05 was considered statistically significant. 

## Results

A total of 80 medico-legal autopsy cases were examined in the present study. The age of the subjects ranged from 21 to 80 years. The descriptive statistics for age, stature, CSL, SSL, and LSL are depicted in Table 1.

**Table 1:** Descriptive statistics of age, stature, CSL, SSL, LSL CSL: Coronal suture length; SSL: Sagittal suture length; LSL: Lambdoid suture length

Parameter	Sex	Mean	SD	Minimum	Maximum
Age (Years)	Male	41.39	17.04	21	80
	Female	27.50	9.86	21	55
	Combined	36.87	16.38	21	80
Stature (mm)	Male	1698.70	80.91	1440	1840
	Female	1585.38	92.92	1400	1810
	Combined	1661.87	99.89	1400	1840
CSL (mm)	Male	160.37	16.91	127.82	206.20
	Female	155.90	23.44	124.11	207
	Combined	158.91	19.24	124.11	207
SSL (mm)	Male	140.12	13.81	100.48	186.88
	Female	132.97	12.03	111.95	156.52
	Combined	137.80	13.61	100.48	186.88
LSL (mm)	Male	178.31	17.73	143.07	207.77
	Female	162.57	19.74	136.04	191.64
	Combined	173.19	19.73	136.04	207.77

Comparison of stature between genders using an independent sample t-test was performed, which indicated a significant statistical difference between the two groups with p-value of 0.0005 (Table 2).

**Table 2:** Comparison of stature (mm) between genders by Independent sample t-test **: Statistically significant at p < 0.01 level

Variable	Gender	N	Mean	SD	t-value	p-value
Stature (mm)	Male	54	1698.7	80.9	5.588	0.0005**
	Female	26	1585.4	92.9		

Table 3 presents a comparison of CSL measurements between male and female subjects. Since the p-value exceeds the conventional threshold of 0.05, the results indicate that there is no statistically significant difference in CSL between male and female subjects in this sample.

**Table 3:** Comparison of CSL (mm) between genders by Independent sample t-test #: No statistical significance at p > 0.05 level CSL: Coronal suture length

Variable	Gender	N	Mean	SD	t-value	p-value
CSL(mm)	Male	54	160.4	16.9	0.870	0.390^#^
	Female	26	155.9	23.4		

A t-test was conducted to assess the statistical significance of the difference in SSL between the two groups, yielding a t-value of 2.255 which suggested that the difference in means is notable, and the p-value of 0.027 indicates that this difference was statistically significant (Table 4).

**Table 4:** Comparison of SSL (mm) between genders by Independent sample t-test *: Statistically significant at p < 0.05 level SSL: Sagittal suture length

Variable	Gender	N	Mean	SD	t-value	p-value
SSL (mm)	Male	54	140.1	13.8	2.255	0.027*
	Female	26	133.0	12.0		

Table 5 presents the findings from a statistical comparison of LSL between male and female subjects. The p-value of 0.001 indicates a high level of statistical significance, as it is well below the 0.01 threshold.

**Table 5:** Comparison of LSL (mm) between genders by Independent sample t-test **: Statistical significance at p < 0.01 level LSL: Lambdoid suture length

Variable	Gender	N	Mean	SD	t-value	p-value
LSL (mm)	Male	54	178.3	17.7	3.583	0.001**
	Female	26	162.6	19.7		

The correlations between stature and the lengths of the coronal, sagittal, and lambdoid sutures in the combined group were analyzed using Pearson correlation coefficients. The results indicate a positive correlation between stature and coronal length, but it was not statistically significant at the 0.05 level. In contrast, the correlations with SSL and LSL show slightly stronger associations with stature (Table 6).

**Table 6:** Correlations of stature (mm) with CSL< SSL, and LSL (mm) by using Pearson correlation **: Significant at p < 0.01; #: No statistical significance at p > 0.05 level CSL: Coronal suture length; SSL: Sagittal suture length; LSL: Lambdoid suture length

Correlations				
		CSL (mm)	SSL (mm)	LSL (mm)
Stature (mm)	r-value	0.212	0.302**	0.297**
	p-value	0.059^#^	0.006**	0.007**

The correlations between stature (in millimeters) and the length of coronal, sagittal and lambdoid suture in male and female subjects was done using Pearson correlation coefficients. The results indicated that the correlations with CSL, SSL, and LSL in male and female subjects were not statistically significant (Tables 7, 8).

**Table 7:** Correlations of stature (mm) with CSL, SSL, and LSL (mm) in male subjects by using Pearson correlation #: No statistical significance at p > 0.05 level CSL: Coronal suture length; SSL: Sagittal suture length; LSL: Lambdoid suture length

Correlations				
		CSL (mm)	SSL (mm)	LSL (mm)
Stature (mm)	r-value	0.060	0.241	0.154
	p-value	0.666^#^	0.080^#^	0.266^#^

**Table 8:** Correlations of stature (mm) with CSL, SSL, and LSL (mm) in female subjects by using Pearson correlation #: No statistical significance at p > 0.05 level CSL: Coronal suture length; SSL: Sagittal suture length; LSL: Lambdoid suture length

Correlations				
		CSL (mm)	SSL (mm)	LSL (mm)
Stature (mm)	r-value	0.349	0.143	0.072
	p-value	0.081^#^	0.487^#^	0.728^#^

A simple regression analysis was aimed at predicting stature (mm) in combined group based on SSL. The regression equation derived from the analysis is:

Stature (mm) = 1356.151 + SSL (mm) x 2.219

This equation implies that with each millimeter increase in sagittal length, stature is predicted to increase by approximately 2.219 mm. This positive association is further reinforced by the statistical significance of the relationship, evidenced by a p-value of 0.006, which is significant. The R^2^ value of 0.091 indicates that around 9.1% of the variance in stature can be explained by SSL. Although statistically significant, the regression model explains only a small proportion of the variance in stature. Additionally, the 95% confidence interval for the coefficient of SSL ranges from 0.641 to 3.796, which does not include zero. This further reinforces the idea that SSL has a positive effect on stature, providing a limited evidence for considering it a weak predictor. SSL demonstrates a statistically significant but weak predictive relationship with stature (Table 9).

**Table 9:** Linear regression analysis for stature in combined group by length of sagittal suture **: Statistical significance at p < 0.01 level SSL: Sagittal suture length; LB: Lower bound; UB: Upper bound

R	R^2^	Adjusted R^2^
0.302	0.091	0.080

Coefficients							
	Unstandardized Coefficients		Standardized Coefficients	t	p-value	95.0% Confidence Interval for B	
	B	Std Error	Beta			LB	UB
(Constant)	1356.151	109.696		12.363	0.000	1137.763	1574.540
SSL (mm)	2.219	0.792	0.302	2.800	0.006**	.641	3.796

A simple regression analysis was aimed at predicting stature (mm) in combined group based on LSL (mm). The regression equation derived from the analysis is:

Stature (mm) = 1401.116 + LSL (mm) x 1.506

The correlation coefficient (R) is 0.297, indicating a weak positive correlation between the two variables, suggesting that as LSL increases, stature tends to increase slightly. However, the R² value is 0.088, meaning that only approximately 8.8% of the variance in stature can be explained by LSL, indicating limited predictive capability of the regression model. Statistical significance is highlighted by a p-value of 0.007 for LSL, which is less than the 0.01 threshold, demonstrating a significant but weak association. This significance suggests that the observed relationship is unlikely to be due to random chance. Furthermore, the 95% confidence interval for the LSL coefficient ranges from 0.416 to 2.595, indicating that 95% confidence interval lies within this range (Table 10).

**Table 10:** Linear regression analysis for stature in combined group by length of lambdoid suture **: Statistical significance at p < 0.01 level LSL: Lambdoid suture length; LB: Lower bound, UB: Upper bound

R	R^2^	Adjusted R^2^
0.297	0.088	0.077

Coefficients							
	Unstandardized Coefficients		Standardized Coefficients	t	p-value	95.0% Confidence Interval for B	
	B	Std Error	Beta			LB	UB
(Constant)	1401.116	95.378		14.690	0.000	1211.234	1590.999
LSL (mm)	1.506	0.547	0.297	2.751	0.007**	0.416	2.595

Table 11 compares known and estimated statures along with results from a paired t-test. The paired t-test results reveal that the mean differences between known and estimated statures are minimal in the present study (Table 12). P-value of 0.972 for the combined group indicated that these difference was not statistically significant. This finding suggests the absence of systematic bias between measured and estimated stature; however, the relatively low R² values indicate that the regression models possess limited predictive precision.

**Table 11:** Descriptive statistics of mean values of known and estimated stature

Parameter	Sex	Number	Minimum	Maximum	Mean	SD
Known stature	Combined Sex	80	1400	1840	1661.87	99.89
Estimated stature	Combined Sex	80	1595.24	1767.98	1662.24	35.22

**Table 12:** Paired t-test between known and estimated stature

Parameter	Sex	Mean	t	Sig
Estimated stature	Combined Sex	-0.363	-0.035	0.972

## Discussion

Forensic identification frequently relies on reconstruction of a biological profile from skeletal remains, and stature estimation constitutes a crucial component of this profile. While long bones provide the most reliable estimates of stature, such elements are not always available in forensic contexts involving fragmentation, dismemberment, or advanced decomposition. In such circumstances, alternative skeletal indicators, particularly cranial parameters, may provide supportive information for estimating stature when conventional skeletal elements are absent. The present study evaluated the relationship between cranial suture lengths and stature in a North Indian population using an autopsy-based morphometric approach. The low R² values observed in the present study indicate limited explanatory power and restricted standalone forensic applicability of the regression models.

In the combined sample, sagittal and lambdoid sutures demonstrated weak but statistically significant correlations with stature, whereas the coronal suture exhibited a weaker and non-significant association. However, when men and women were analyzed separately, the correlations between stature and the three sutures were not statistically significant. The absence of statistically significant sex-specific correlations suggests that the pooled associations may partly reflect underlying sexual dimorphism rather than a direct biological relationship between cranial suture length and stature.These findings indicate that cranial suture length alone is a relatively weak predictor of overall body stature as limited associations were observed when the population is analyzed as a combined group. The relatively weak correlations observed in the present study may be explained by the fact that cranial vault growth and overall body stature are influenced by different developmental and genetic factors. Stature is primarily determined by the longitudinal growth of long bones through epiphyseal plate activity, whereas cranial sutures mainly reflect cranial vault expansion associated with brain growth and cranial morphology. Because these developmental processes are regulated by different genetic and environmental factors, cranial suture length may show only limited association with overall body height. Therefore, cranial suture measurements should not be regarded as primary predictors of stature. Instead, they may serve as auxiliary parameters in forensic investigations, particularly in situations where long bones are unavailable due to fragmentation, dismemberment, or advanced decomposition.

The findings of the present study show both similarities and differences when compared with previous studies. Razdan et al. reported a moderate correlation between supine length and CSL (r = 0.324) in the combined group, with statistically significant correlations also observed separately in male and female subjects [11]. Similarly, Tiwari et al. observed a moderate correlation between stature and CSL in the combined group (r = 0.416), while the correlations for sagittal sutures were comparatively weaker but statistically significant [12]. In contrast, Kolencherry et al. reported very weak and statistically non-significant correlations between SSL and CSL and stature in a Central European population [13]. Venkatesh et al. demonstrated significant positive correlations between stature and both CSL and SSL in their cadaveric study conducted in South India [10]. Conversely, Rao et al. reported a statistically significant correlation between stature and CSL but observed no significant relationship with SSL [14]. Variability among studies may also reflect differences in cranial morphology associated with genetic background, environmental influences, nutrition, and regional population characteristics. Additionally, methodological differences including measurement techniques, age composition, and extent of suture complexity or obliteration may contribute to inconsistent findings across studies.Since stature estimation is known to be population-specific, regression equations derived in one population may not be directly applicable to another. The comparative analysis of correlation coefficients from previous studies and the present study is summarized in Table 13, which illustrates the variability in correlation strength reported across different populations and research settings.

**Table 13:** Comparison of correlation coefficients of various studies in combined group *: No statistical significance at p > 0.05 level

Sr. No.	Study	Sample size	Correlation coefficient		
			CSL	SSL	LSL
1	Present study	80	0.212*	0.302	0.297
2	Venkatesh et al. [10]	210	0.326	0.308	-
3	Razdan et al. [11]	150	0.324	-	-
4	Tiwari et al. [12]	500	0.416	0.251	0.082*
5	Kolencherry et al. [13]	117	0.015*	0.045*	-

In the present study, linear regression models based on sagittal and lambdoid sutures achieved statistical significance but demonstrated relatively coefficients of determination (R² values). The low R² values (<0.10) indicate that cranial suture lengths explain only a small proportion of stature variability and therefore possess limited standalone forensic utility.These findings suggest that only a small proportion of variation in stature can be explained by these cranial measurements. Therefore, cranial suture lengths should be regarded as supplementary indicators rather than primary predictors of stature in forensic practice. Their value lies mainly in situations where other skeletal elements-particularly long bones-are unavailable.

Table 14 presents a comparison of the standard error and coefficient of determination (R²) values reported in previous studies. The relatively low R² values observed in the present study further support the limited predictive capability of cranial suture measurements when used in isolation. The standard error of estimate values observed in the present study further indicate relatively broad prediction error ranges, limiting precision in practical forensic applications.

**Table 14:** Comparison of standard error and R2 of various studies

Sr. No.	Study	Name of Suture	Gender	Std Error	R^2^
1	Present study	Sagittal	Combined	0.792	0.091
		Lambdoid	Combined	0.547	0.088
2	Razdan et al. [11]	Coronal	Male	109.720	-
		Coronal	Female	62.04	-
		Coronal	Combined	95.03	-
3	Tiwari et al. [12]	Coronal	Male	0.44	-
		Coronal	Female	0.60	-
		Coronal	Combined	0.37	-
		Sagittal	Male	46.940	-
		Sagittal	Female	40.016	-
		Sagittal	Combined	63.439	-
4	Venkatesh et al. [10]	Coronal	Female	6.0271	0.09
		Coronal	Combined	8.302	0.106
		Sagittal	Combined	5.194	0.095
6	Rao et al. [14]	Coronal	Male	5.67	-

The paired t-test results in the present study revealed that the mean difference between known and estimated statures was minimal, and the difference was not statistically significant (p = 0.972). This suggests that although the regression equations do not introduce systematic estimation bias, their predictive precision remains limited due to the weak correlations between the variables.

Despite the modest predictive ability observed in this study, cranial suture measurements remain advantageous because they can be obtained even when cranial fragments are incomplete. When combined with other cranial metrics, they may contribute to a multivariate model that improves stature estimation accuracy.

Limitations

Several limitations of the present study should be acknowledged. First, the sample size was relatively small with unequal sex distribution, which may have reduced statistical power, particularly for female subgroup analyses. Second, the regression models demonstrated low explanatory power with limited predictive accuracy. Third, formal intraobserver and interobserver reliability testing was not performed, and minor measurement variability may have occurred due to manual thread placement along irregular cranial sutures. Fourth, pooled-sex analysis may have introduced partial confounding due to sexual dimorphism. Fifth, external or internal validation analyses were not performed, limiting assessment of reproducibility and generalizability. Finally, supine postmortem body length may differ slightly from living stature because of postmortem physiological changes. Therefore, the derived equations should be regarded as exploratory and supplementary rather than definitive forensic tools.

## Conclusions

The present study demonstrated statistically significant but weak associations between stature and sagittal as well as lambdoid cranial suture lengths in a North Indian population. However, the low coefficients of determination and limited predictive accuracy indicate that cranial suture measurements possess restricted forensic applicability when used independently. These parameters should therefore be regarded only as supplementary indicators for stature estimation in situations where conventional skeletal elements are unavailable. Further studies involving larger, sex-balanced, and multicentric populations with validation analyses are necessary before practical forensic application can be recommended.
